# The RING Domain of Rice HEI10 is Essential for Male, But Not Female Fertility

**DOI:** 10.1186/s12284-023-00681-w

**Published:** 2024-01-05

**Authors:** Qian Tan, Xu Zhang, Qian Luo, Yi-Chun Xu, Jie Zhang, Wan-Qi Liang

**Affiliations:** Joint International Research Laboratory of Metabolic and Developmental Sciences, State Key Laboratory of Hybrid Rice, School of Life Sciences and Biotechnology, Shanghai Jiao Tong University, Shanghai, China

**Keywords:** HEI10, Rice, Meiosis, Fertility, Gamete, Heterochiasmy, RING domain

## Abstract

HEI10 is a conserved E3 ubiquitin ligase involved in crossover formation during meiosis, and is thus essential for both male and female gamete development. Here, we have discovered a novel allele of *HEI10* in rice that produces a truncated HEI10 protein missing its N-terminal RING domain, namely *sh1* (*shorter hei10 1*). Unlike previously reported *hei10* null alleles that are completely sterile, *sh1* exhibits complete male sterility but retains partial female fertility. The causative *sh1* mutation is a 76 kb inversion between *OsFYVE4* and *HEI10*, which breaks the integrity of both genes. Allelic tests and complementation assays revealed that the gamete developmental defects of *sh1* were caused by disruption of *HEI10*. Further studies demonstrated that short HEI10 can correctly localise to the nucleus, where it could interact with other proteins that direct meiosis; expressing short HEI10 in *hei10* null lines partially restores female fertility. Our data reveal an intriguing mutant allele of *HEI10* with differential effects on male and female fertility, providing a new tool to explore similarities and differences between male and female meiosis.

**Supplementary Information:**

The online version contains supplementary material available at 10.1186/s12284-023-00681-w.

## Background

An essential step in sexual reproduction is the production of haploid gametes via meiosis, a complex process in which one round of chromosome replication is followed by two rounds of cell division (meiosis I and meiosis II). During meiosis I, homologous chromosomes establish physical links via homologous recombination, which is vital for faithful chromosome separation and to generate genetic diversity in progeny. Several key events, such as synaptonemal complex (SC) assembly and crossover (CO) formation, occur to ensure proper progression of meiosis. Two types of COs are produced during the repair of double-stranded DNA breaks. Of these, class I COs in plants are produced by a set of conserved meiotic-specific ZMM proteins named after yeast Zip, Msh, and Mer proteins (Rockmill et al. 2003; Fung et al. 2004; Börner et al. 2004), which include HEI10 (Lynn et al. 2007; Mercier et al. 2015).

HEI10 was first identified in humans (Human enhancer of invasion-10), encoding a ubiquitin E3 ligase that plays important roles in cell migration and invasion (Toby et al. 2003; Singh et al. 2007). The HEI10 protein contains an N-terminal RING domain (a C3HC4 zinc finger) that recruits ubiquitin-linked E2 to substrates, while the remainder of the protein contributes to substrate recognition (Deshaies and Joazeiro 2009). HEI10 and its homologues are widely distributed in almost all known species. Only a single HEI10 orthologue is present in the genome of plants, prokaryotes, and fungi, whereas mammals contain not only HEI10 but also its antagonistic SUMO E3 ligase RNF212 (Ward et al. 2007; Bhalla et al. 2008; Chelysheva et al. 2012; Wang et al. 2012; Reynolds et al. 2013; Serrentino et al. 2013; De Muyt et al. 2014; Qiao et al. 2014; Lake et al. 2015; Gray and Cohen 2016; Rao et al. 2017). In plants, Arabidopsis and rice (*Oryza sativa*) *HEI10* are thought to be homologues of yeast (*Saccharomyces cerevisiae*) *ZIP3*, which specifically promotes class I CO formation through modification of various meiotic components. Rice *HEI10* has been shown to play an important role in CO formation during meiotic recombination (Wang et al. 2012).

Mutations that affect meiosis usually affect the development of both male and female gametes (Wang et al. 2012; Zhang et al. 2019). However, a few meiosis-related mutations have been reported to disrupt only male fertility. In rice, *OsPSS1* encodes a kinesin-1-like protein essential for male meiotic chromosomal dynamics and gametogenesis, while female fertility is unaffected (Zhou et al. 2011). *OsMIL1* plays an important role in meiotic entry, mutation of which leads to failure of male meiosis initiation in anthers while female gametes develop normally (Hong et al. 2012). Recently, mutation of a central component of the meiotic SC, *OsZEP1* in the background of a hybrid rice CY84, was found to cause only male sterility, which provides a potential avenue to increase genetic diversity and break linkage drag in rice breeding (Liu et al. 2021). Despite the clear differences in male and female fertility caused by mutation of these meiosis-related genes, they are not specifically expressed in the male reproductive organs; understanding of their underlying mechanisms of action remains elusive.

Heterochiasmy refers to differences in the frequency of recombination rate in male and female gametes during meiosis, including quantitative discrepancy and location of COs (Burt et al. 1991; Lenormand 2003; Lenormand and Dutheil 2005; Drouaud et al. 2007). This phenomenon is widely observed in many species, including humans, mice, and Arabidopsis (Morelli and Cohen 2005; Petkov et al. 2007; Gruhn et al. 2016; Durand et al. 2022). In Arabidopsis, the average number of COs in female meiocytes is significantly lower than that in male meiocytes (Giraut et al. 2011; Capilla-Pérez et al. 2021). It is proposed that heterochiasmy originated via several mechanisms, such as epistatic interactions (Lercher and Hurst 2003), X-linked modifiers (de la Casa-Esperón et al. 2002), chromatin structure differences (Gerton and Hawley 2005), and haploid selections (Lenormand and Dutheil 2005). The intensity of CO interference and SC length are thought to influence heterochiasmy (Kleckner et al. 2003; Shang et al. 2022). RNF212 is highly associated with heterochiasmy in animals (Kong et al. 2014; Johnston et al. 2016), while HEI10 is proposed to affect CO number and heterochiasmy in a dose-dependent manner in Arabidopsis (Capilla-Pérez et al. 2021; Morgan et al. 2021; Durand et al. 2022).

In this study, we report the identification in rice of a novel allele of the vital ZMM family protein, HEI10, named *sh1* (*shorter hei10 1*). The mutant expresses a truncated HEI10 protein missing its N-terminus RING domain, and exhibits a different phenotype to the completely sterile *hei10* null mutant; *sh1* retains partial female fertility but remains male-sterile. We also show that the truncated HEI10 is able to interact with other proteins involved in CO formation, and correctly localises to the nucleus. Furthermore, we demonstrate that expressing short *HEI10* in the *hei10* knockout allele can partially restore female fertility. These results indicate that the RING domain of HEI10 is essential for male but not female meiosis, suggesting a role for this domain in heterochiasmy.

## Materials and Methods

### Plant Materials and Growth Conditions

Rice (*Oryza sativa*) plants were grown in the paddy fields of Shanghai Jiao Tong University (30 °N 121 °E) according to standard local practice during the natural growing season (June–September). *O. sativa* ssp. *japonica* 9522 was used as the wild-type. The *hei10* line was described previously (Wang et al. 2017).

### Phenotypic Characterisation

A Nikon E995 digital camera was to image whole rice plants and panicles. Flowers were photographed with a Leica M205A microscope. For pollen viability analysis, anthers were immersed into Lugol’s iodine (I_2_–KI) solution and crushed with tweezers; released pollen grains were photographed with a Nikon Eclipse 80i microscope.

Transverse section analysis of developing anthers was described previously (Li et al. 2006). For scanning electron microscopic (SEM) observations, flowers were fixed in FAA solution (5 ml 38% formaldehyde, 5 ml acetic acid, 50 ml ethanol and 40 ml ddH_2_O for 100 ml FAA solution) overnight and dehydrated in a 60%, 70%, 80%, 90% and 100% ethanol series. The samples were dried using a Leica EM CPD300 automated critical dryer, and coated with gold using a cool sputter coater (Leica EM SCD005). Anther and pollen grain surfaces were photographed using a Hitachi S3400N SEM.

### Cloning of *sh1* and Complementation Test

The *sh1* mutant was identified from a mutant population generated and described by (Liu et al. 2005). The F_2_ mapping population was generated from a cross between the *sh1* mutant (*japonica*) and Guangluai 4 (wild-type, *indica*). Male sterile plants in the F_2_ progeny were selected for gene mapping. To fine-map the *sh1* locus, bulked segregant analysis was used, and insertion-deletion (indel) molecular markers were designed on the basis of the sequence differences between *japonica* and *indica* described in the NCBI database. The *sh1* locus was first mapped between two indel molecular markers: Chr2_1444 and Chr2_2629. Then, 101 F_2_ segregants from the mapping population and eight indel markers were used for fine mapping. *sh1* was eventually located between C2 and C4 within a 245 kb region. Primers used in the mapping are listed in Additional file 2: Table S1.

For complementation test, a 7788 bp genomic sequence containing the entire coding region of rice *HEI10* (2092 bp), a 4899 bp upstream sequence, and a 797 bp downstream sequence was amplified using wild-type rice genomic DNA and cloned into the binary vector pCAMBIA1301. Primers for amplification are given in Additional file 2: Table S1. This construct was transformed into *sh1* calli to create the *Hei10-gDNA;sh1* complementation lines.

### Generation and Selection of *sh1/hei10* Biallelic Lines

Pollens from heterozygous *HEI10/hei10* plants were pollinated on homozygous *sh1* pistils, and F_1_ plants were selected by PCR identification and sequencing. A pair of primers (*ID-sh1-F* and *ID-sh1-R*) were designed to identify the chromosome containing *sh1* locus, while *ID-hei10-F* and *ID-sh1-R* were used to monitor the wild-type or *hei10* allele. Primers used in this are shown in Additional file 2: Table S1.

## Generation of CRISPR Knockout Mutants, *SH1-gDNA* and *Ubi:SH1cds* Transgenic Plants

CRISPR-Cas9 mutants were obtained using methods described previously (Wang et al. 2017). The primers for knockout constructs and mutant line identification are shown in Additional file 2: Table S1.

To create the short *HEI10* genomic DNA construct (*SH1-gDNA*), a 8575 bp genomic sequence was amplified from *sh1* genomic DNA, comprising the 6018 bp sequence upstream of the third *HEI10* exon plus the 2557 bp sequence from the start of the third exon, and cloned into the binary vector pCAMBIA1301. To create the maize ubiquitin promoter–driven short *HEI10* construct (*Ubi:SH1cds*), the coding sequence from the second start codon to the stop codon of short *HEI10* was amplified from *sh1* cDNA, and inserted into binary vector pTCK303 downstream of maize ubiquitin promoter. These constructs were then transformed into *hei10* calli to create the *SH1-gDNA;hei10* and *Ubi:SH1cds;hei10* transgenic lines, as described previously (Wang et al. 2017). Primers used in this are shown in Additional file 2: Table S1.

### Chromosome Spreads and Immunofluorescence Analysis

For DAPI staining, young panicles at developmental stages when male meiosis is occurring were fixed in Carnoy’s solution (3:1 ethanol:acetic acid) for at least 24 h. Anthers were picked out with a needle and crushed with tweezers in 1% (w/v) acetocarmine and covered with a coverslip. Slides were frozen in liquid nitrogen and the coverslips removed. Chromosomes were then stained with 4′,6-diamidino-2-phenylindole (DAPI) solution and imaged as described previously (Cheng 2013).

For immunolocalisation analysis, young panicles at developmental stages when male meiosis is occurring were fixed in 4% (w/v) paraformaldehyde for 30 min at room temperature. Anthers were picked out with a needle and crushed with tweezers in phosphate-buffered saline (PBS) and covered with a coverslip. Slides were frozen in liquid nitrogen and the coverslips removed. Slides were then incubated in a humid chamber at 37 ℃ for 4 h with anti-HEI10 (rabbit) and anti-REC8 (rat) polyclonal antibodies (diluted 1:500 in TNB buffer [0.1 M Tris-HCl, 0.15 M NaCl, pH 7.5, and 0.5% [w/v] blocking reagent]; antibodies generated as described previously (Zhang et al. 2019). After three rounds of washing in PBS, Alexa 555-conjugated goat anti-rabbit antibody (Life Technologies; 1:200) or DyLight488-conjugated goat anti-rat antibody (Abbkine; 1:200) were added to the slides. Chromosomes were counterstained with DAPI solution. Fluorescence was captured using an Eclipse Ni-E microscope (Nikon), and analysis was performed using NIS-Elements Advanced Research software. Image deconvolution was carried out using the function “Mexican Hat.”

### Subcellular Localisation Analysis

The coding region of short HEI10 was cloned into pHB-35S_pro_-eGFP and transformed into *A. tumefaciens* GV3101. Briefly, bacteria were collected and resuspended in infection buffer (10 mM MES, 10 mM MgCl_2_ and 200 µM acetosyringone, A_600nm_ = 0.6), then infiltrated into *Nicotiana benthamiana* leaves and grown in the dark for 48 h. eGFP fluorescence was captured by a Leica SP5 confocal microscope (excitation 488 nm, emission 500–555 nm). Primers used in this are shown in Additional file 2: Table S1.

### Cytological Analysis of Embryo Sacs

Mature ovaries were harvested and fixed in Carnoy’s solution overnight, and rehydrated with a 50%, 30%, 15% ethanol series and absolute water. Samples were incubated in 1 M HCl at 60℃ for 15 min, and pre-stained with 1% eosin Y dissolved in ethanol for 8 h. After several rounds of washing with distilled water, samples were transferred into a solution containing 0.1 M citric acid and 0.2 M disodium hydrogen phosphate (pH 5.0) overnight. The samples were further stained by Bisbenzimide Hoechst 33,342 (20 µg ml^−1^) at 25℃ for 24 h. After 3 rounds of washing with distilled water, samples were dehydrated with a 15%, 30%, 50%, 70%, 85%, 95%, and 100% ethanol series overnight. Finally, samples were incubated in 1:1 methyl salicylate:ethanol for 1 h, and then stored in 100% methyl salicylate until observation. Images of embryo sacs were captured using a Leica SP5 confocal microscope (excitation 488 nm, emission 500–555 nm).

### Yeast Two-Hybrid (Y2H) Assay

The coding sequence of rice *HEIP1*, full-length *HEI10*, and short *HEI10* were amplified and cloned into pGADT7 and pGBKT7 (Clontech) and transformed into yeast strain AH109. Y2H constructs of *PTD*, *ZIP4*, and *MSH5* were described previously (Zhang et al. 2019). Subsequent Y2H assays were performed with the Matchmaker Gold Yeast Two-Hybrid System according to the manufacturer’s instructions (Clontech). In brief, the indicated construct combination was transformed into AH109 and plated on − 2 SD (SD medium without Lue and Trp) agar plates. The positive clones were resuspended in ddH_2_O and transferred on − 3 SD (without Lue, Trp and His) and − 4 SD (without Lue, Trp, His and Ade) agar plates to test the protein interaction. Primers used in this are shown in Additional file 2: Table S1.

### Immunoblotting of HEI10

Total protein was extracted from spikelets at Stage 7–8 from wild-type, *sh1*, *hei10* and *SH1gDNA:hei10* lines by boiling for 5 min in 2× SDS-PAGE loading buffer (100 mM Tris-HCl, 4% w/v SDS, 2% w/v bromophenol blue, 20% v/v glycerol, 2% v/v 14.4 M 2-mercaptoethanol, pH6.8) and analysed by immunoblotting using anti-HEI10 antibody (1:1000 dilution) described above. Anti-tubulin antibody (Abmart, M20045 1:5000 dilution) was used as a internal control.

### Accession Numbers

Sequence data from this article can be found in the Rice Genome Annotation Project (http://rice.plantbiology.msu.ed) under accession numbers: *OsHEI10* (LOC_Os02g13810), *OsFYVE4* (LOC_Os02g13890), *OsHEIP1* (LOC_Os01g07330), *OsPTD* (LOC_Os05g51060), *OsZIP4* (LOC_Os01g66690), *OsMSH5* (LOC_Os05g41880).

## Results

### Characterization of a Male Sterile Mutant *sh1*

**Fig. 1:**
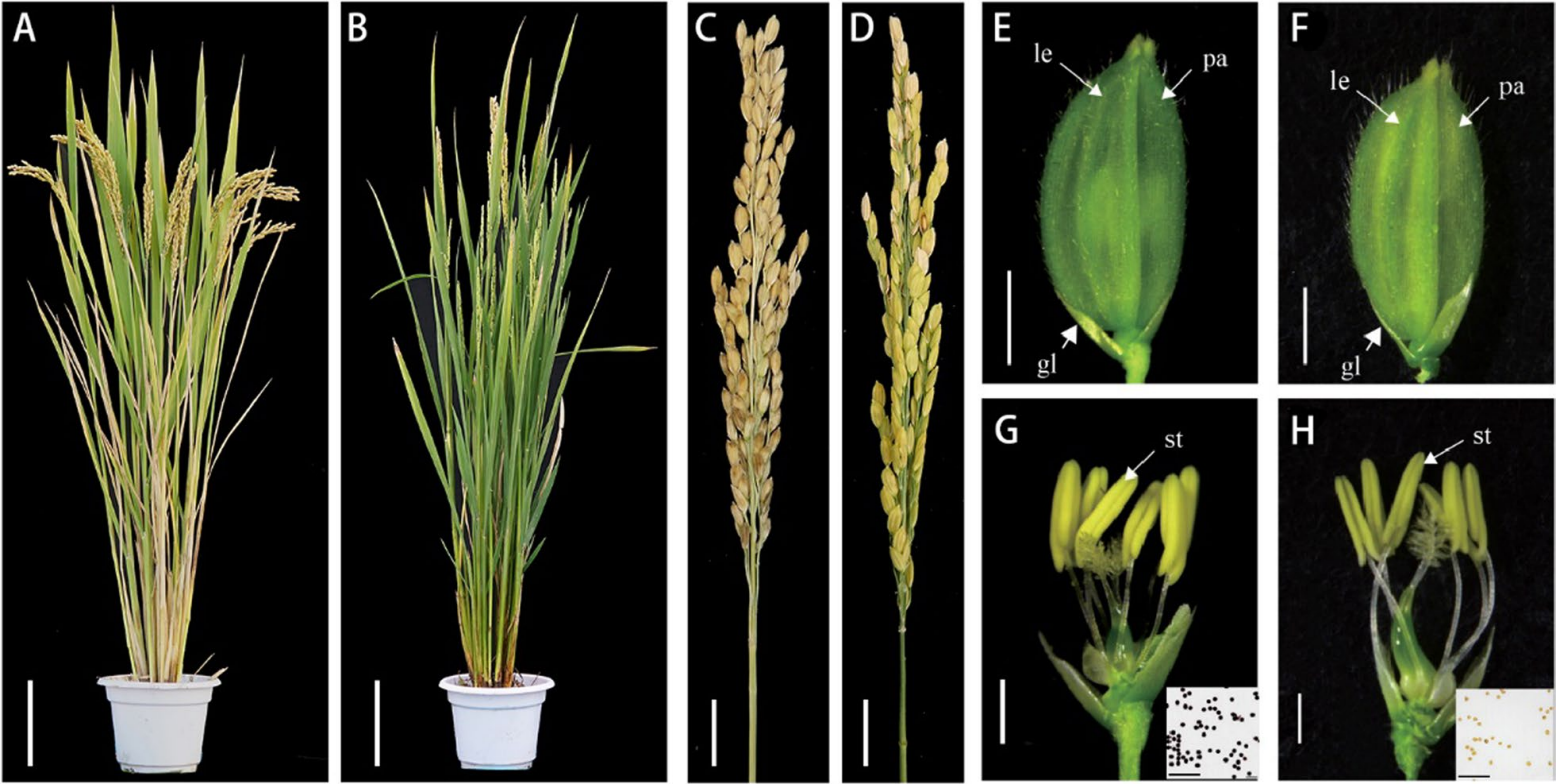
Phenotype of *sh1* mutant. A wild-type (**A**) and *sh1* (**B**) plant after heading. Scale bars = 10 cm. A wild-type (**C**) and *sh1* (**D**) panicle. Scale bars = 1 cm. A wild-type (**E**) and *sh1* (**F**) spikelet at maturity. Scale bars = 1 mm. A wild-type (**G**) and *sh1* (**H**) flower at maturity after removing the palea (p.a.) and lemma (le). Scale bars = 1 mm. Insets show I_2_–KI staining of mature pollen grains from the stamen (st); dark blue staining indicates viable pollen. Scale bars = 0.3 mm

A rice male sterile mutant *sh1* was isolated from our mutant library in *O. sativa* ssp. *japonica* 9522 (Chen 2006). The mutant showed normal vegetative growth (Additional file 1: Fig. S1) but was sterile at maturity (Fig. 1A–D). The *sh1* floral organs showed no defect compared to the wild-type (WT), except for thinner anthers containing no viable pollen (Fig. 1E–H). When pollinated with WT 9522 pollen, *sh1* was able to set seed. All F_1_ progeny were fertile, but the F_2_ population displayed an approximate 3:1 ratio of fertile to sterile plants (138:47), indicating that *sh1* is a single recessive mutation conferring male, but not female, sterility.

Transverse section analysis of mutant and WT anthers during development revealed cytological defects of *sh1* microspore development (Additional file 1: Fig. S2); anther development stages are as defined by Zhang et al. (2011). Before meiosis, the WT anther cells differentiate into four anther wall cell layers (epidermis, endothecium, middle layer, and tapetum) and the inner microspore mother cells (MMCs). After Stage 7, MMCs undergo two rounds of meiotic division to produce dyads and tetrads (Additional file 1: Fig. S2A, B). At Stages 9 and 10, microspores are released from tetrads and became vacuolated (Additional file 1: Fig. S2C, D), followed by two rounds of mitosis and starch accumulation to form mature pollen at Stage 12 (Additional file 1: Fig. S2E, F). In *sh1* anthers, no obvious morphological defects were found before Stage 12, with normal microsporogenesis and tapetum degradation compared to WT anthers (Additional file 1: Fig. S2G–K). However, mature *sh1* pollen grains were variable in size and not able to accumulate starch (Additional file 1: Fig. S2L).

Scanning electron microscopy (SEM) to observe the surface morphology of the anther epidermis, tapetum, and pollen grains revealed that the anther shape, anther surface, Ubisch bodies on the inner surface of the tapetal layer, and pollen exine wall were similar to WT, but that mutant pollen grains were shrunken (Additional file 1: Fig. S3). These results indicate that *sh1* pollen sterility is not attributable to defects in anther somatic layers, but likely due to the aberrant development of microspores.

### Map-Based Cloning of the *sh1* Locus

**Fig. 2:**
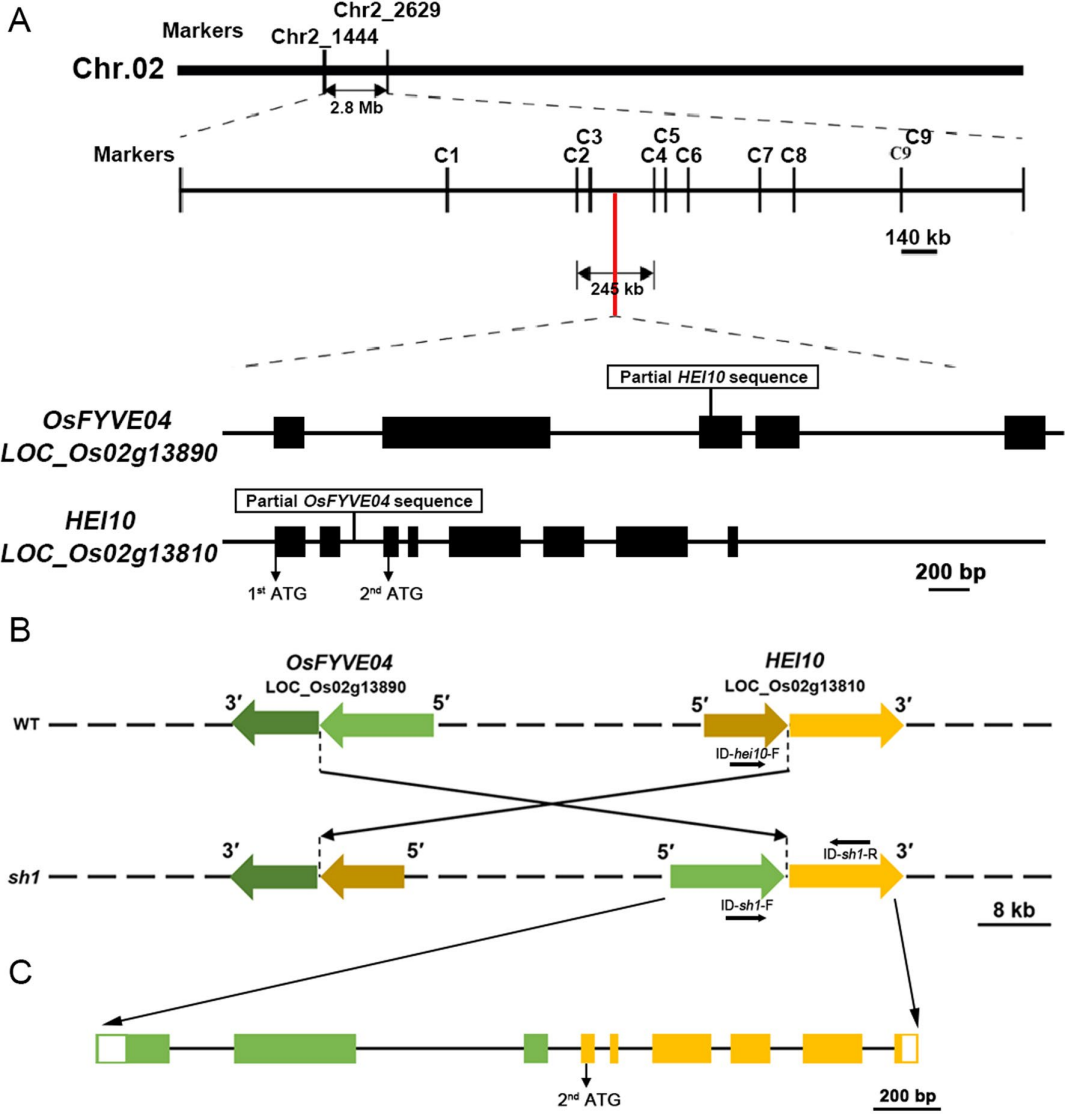
Map-based cloning and analysis of *sh1* locus. **A** Fine mapping of the *sh1* locus on chromosome 2 (top), and schematic representation (bottom) of the exon and intron organisation of *OsFYVE4* (LOC_02g13890) and *HEI10* (LOC_02g13810). Names and positions of the molecular markers are shown. Black boxes indicate exons; intervening lines indicate introns; white squares indicate insertions with relevant genes named. ATG, translational start site. **B** Schematic representation of the 76 kb inversion fragment in *sh1*. Light green and dark green arrows indicate the DNA fragments of *OsFYVE4* outside and inside the inversion, respectively; dark yellow and light yellow arrows indicate the DNA fragments of *HEI10* the DNA fragments inside and outside the inversion, respectively. The primers used for mutant genotyping were indicated by black arrows. **C** Schematic representation of the exon and intron organisation of the recombined *HEI10* locus. Green boxes indicate *OsFYVE4* exons (1, 2, and part of 3); yellow boxes indicate *HEI10* exons (3–8)

A map-based cloning approach was used to identify the locus responsible for the *sh1* phenotype. The mutated gene was located between two markers on chromosome 2, C2 and C4, defining a region of 245 kb containing 31 genes (Fig. 2A). Subsequent analysis of high throughput sequencing indicated a large chromosome fragment (~ 76 kb) inversion between two candidate genes, *OsFYVE04* (LOC_Os02g13890) and *HEI10* (LOC_Os02g13810). One end of the inversion was located in the third exon of *OsFYVE04*, while the other end fit between the second and the third exons of *HEI10* (Fig. 2B, C), which breaks the integrity of both genes.

We first chose to investigate *OsFYVE4* as responsible for the *sh1* phenotype, as the female fertility of *sh1* differed to previously reported female sterility of known *hei10* mutant alleles (Wang et al. 2012). The OsFYVE family has been classified into six groups based on protein domains (Xiao et al. 2016). OsFYVE4 contains a FYVE zinc finger domain in the middle and a C-terminal DUF500/SYLF domain, which places it in group II along with OsFYVE13, OsFYVE15, and OsFYVE17. We used an efficient CRISPR-Cas9 system on *OsFYVE4*, targeting a site in the encoded FYVE domain, and obtained three independent frame-shift mutations (Additional file 1: Fig. S4). No mutant plants displayed phenotypic or fertility changes (data not shown), indicating that the causal gene for the *sh1* phenotype was not *OsFYVE4.*

### *sh1* is a Novel Allele of *hei10*

**Fig. 3:**
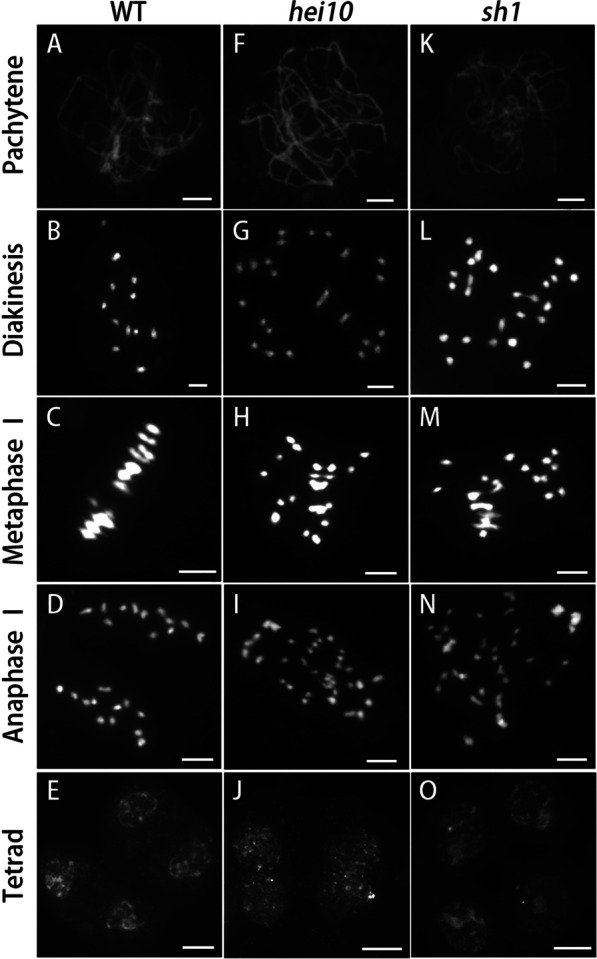
Chromosome behavior in male meiocytes is similar in *hei10* and *sh1* mutants. Chromosome behaviour in wild-type (**A**–**E**), *hei10* (**F**, **J**), and *sh1* (**K**–**O**) male meiocytes at different stages of meiosis: pachytene (**A**, **F**, **K**), diakinesis (**B**, **G**, **L**), metaphase I (**C**, **H**, **M**), anaphase I (**D**, **I**, **N**), and tetrad (**E**, **J**, **O**). Scale bars = 5 μm (**A**–**D**; **F**–**I**; **K**–**N**); 10 μm (**E**, **J**, **O**)

Our focus turned to *HEI10*, whose sequence had been disrupted by deletion of its first and second exons, and the 5′ promoter partially replaced by *OsFYVE4* sequence (Fig. 2C). To verify whether *sh1* sterility was caused by the disruption in *HEI10*, we investigated chromosome behaviour in male meiocytes in WT, *hei10*, and *sh1* lines using DAPI (4′,6-diamidino-2-phenylindole) staining. WT chromosomes condensed into thin threads at leptotene and paired into initial synapsis at zygotene. After the completion of synapsis, the chromosomes appeared as thick threads at pachytene (Fig. 3A). After diplotene, the COs linked homologous chromosomes together and further condensed as 12 clear bivalents at diakinesis (Fig. 3B). At metaphase I, all bivalents aligned along the equatorial plane of the cell (Fig. 3C), and subsequently separated at anaphase I to form dyads (Fig. 3D). Ultimately, after a simultaneous division at meiosis II, the two dyads produced tetrads (Fig. 3E).

In *hei10* male meiocytes, chromosome behaviour was similar to previous reports (Wang et al. 2012). Normally aligned chromosomes were detected at pachytene (Fig. 3F), but both bivalent and univalent chromosomes were found at diakinesis (Fig. 3G). The bivalents were well aligned on the equatorial plate but the univalent chromosomes were randomly scattered in the nucleus (Fig. 3H). Chromosomes underwent asymmetrical migration to opposite poles at anaphase I and formed dyads and tetrads with uneven numbers of chromosomes (Fig. 3I, J). The meiotic progression in *sh1* male meiocytes was nearly identical to that in *hei10* meiocytes, with abnormality appearing at diakinesis resulting in tetrads with aberrant numbers of chromosomes (Fig. 3K–O).

We next assessed the localisation of HEI10 in *sh1* mutant male meiocytes, using co-immunolocalisation with OsREC8. OsREC8 is required for sister chromatid cohesion, axial element formation, and homologue pairing, and is a marker for meiotic chromosomes (Shao et al. 2011). Rice HEI10 was reported to form prominent foci at late prophase I (Wang et al. 2012), but we failed to detect HEI10 foci in most male meiocytes (96.6%) of *sh1*; only a small portion of cells (3.4%) contained HEI10 foci (Additional file 1: Fig. S5). Combining these results of aberrant chromosome behaviour and HEI10 protein localisation in *sh1* lines indicates that *sh1* is likely an allelic mutant of *hei10*.

**Fig. 4:**
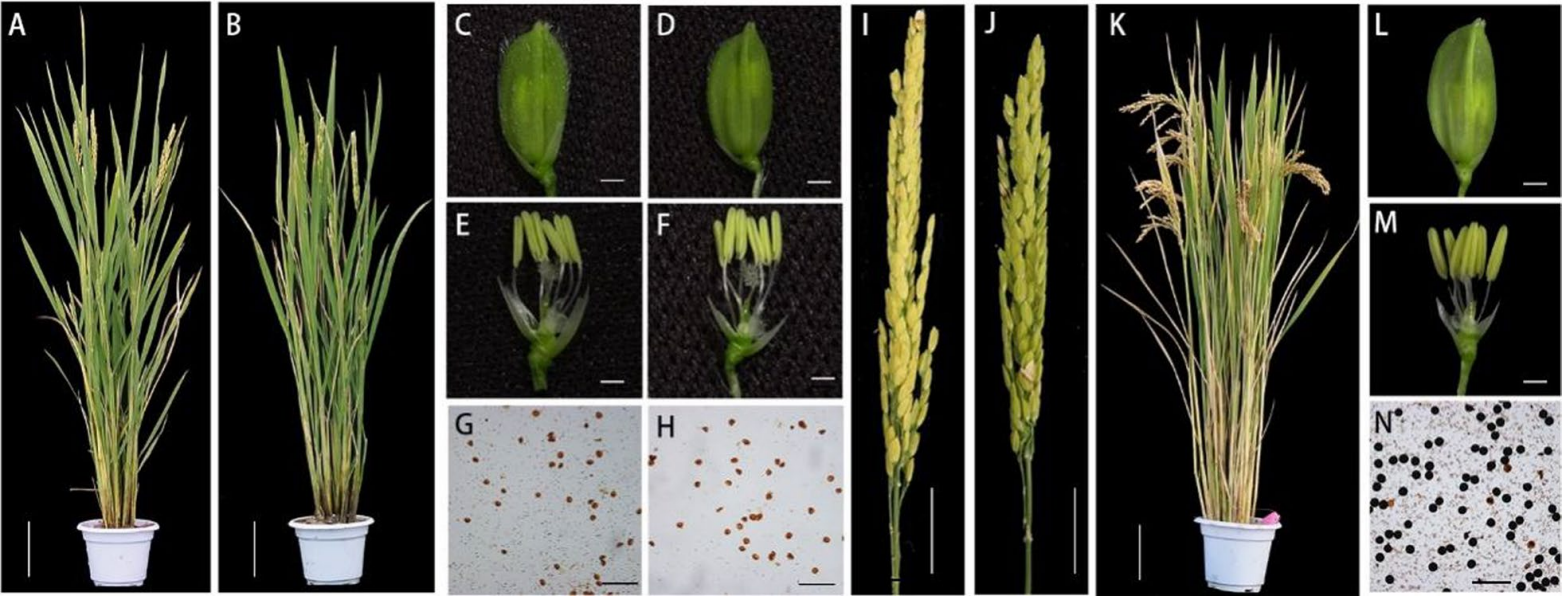
*sh1* is a novel allele of *hei10.* A *hei10* (**A**) and *sh1/hei10* biallelic (**B**) plant after heading. Scale bars = 10 cm. A *hei10* (**C**) and *sh1/hei10* biallelic (**D**) spikelet at maturity. Scale bars = 1 mm. A *hei10* (**E**) and *sh1/hei10* biallelic (**F**) flower after removing the palea and lemma. Scale bars = 1 mm. I_2_–KI staining of *hei10* (**G**) and *sh1/hei10* biallelic (**H**) mature pollen grains. Scale bars = 0.1 mm. A *hei10* (**I**) and *sh1/hei10* biallelic (**J**) panicle at maturity. Scale bars = 5 cm. Mature plant (**K**), spikelet (**L**), flower (**M**), and I_2_–KI stained pollen (**N**) of a *HEI10-gDNA;sh1* complementation line. Scale bars = 1 cm (**K**), 2 mm (**L**, **M**) and 0.2 mm (**N**)

To confirm this hypothesis, we conducted allelic and gene complementation analyses. A homozygous *sh1* mutant was pollinated with pollen grains from *HEI10/hei10* heterozygous plants. We obtained 21 *sh1/hei10* biallelic plants from 48 F_1_ seeds, all of which showed a similar male sterile phenotype to *hei10* and *sh1* homozygous mutants (Fig. 4A–J), while *sh1/HEI10* plants displayed normal fertility (data not shown), suggesting that *sh1* is allelic to *hei10*. This result was further confirmed via genetic complementation, using a 7.8 kb genomic fragment of wild-type *HEI10* (*HEI10-gDNA* including the entire coding region and upstream and downstream regulatory regions) cloned into a binary vector and transformed into *sh1* lines. All eleven transgenic plants displayed WT male fertility and flower organ phenotypes (Fig. 4K–N), demonstrating that the *sh1* mutant phenotype was caused by the mutation of *HEI10* gene.

In *sh1* lines, *HEI10* gene structure is disrupted by a 76 kb genomic inversion (Fig. 2B). Apart from missing the first two exons, the *HEI10* coding sequence was intact (Fig. 2C). A start codon (ATG) occurred at the beginning of the third exon, which may produce a truncated version of HEI10 in *sh1* lines (Fig. 2C). WT HEI10 is 304 aa in length, with a conserved RING domain at its N-terminus (aa 3–41; Additional file 1: Fig. S6). The short *HEI10* (*sHEI10*) coding region, starting at the second start codon, is predicted to encode a 255 aa protein that corresponds to 50–304 aa of full-length HEI10, missing the N-terminal RING domain (Additional file 1: Fig. S6). Furthermore, SWISS-MODEL (https://swissmodel.expasy.org/) analysis showed that absence of the N-terminal RING domain did not obviously affect the protein structure of the remaining part (Additional file 1: Fig. S6). A western-blot assay confirmed a smaller protein detectable by the anti-HEI10 antibody is indeed expressed in *sh1* lines (Additional file 1: Fig. S7).

### The Truncated *HEI10* Partially Restores the Female Fertility of *hei10*

**Fig. 5:**
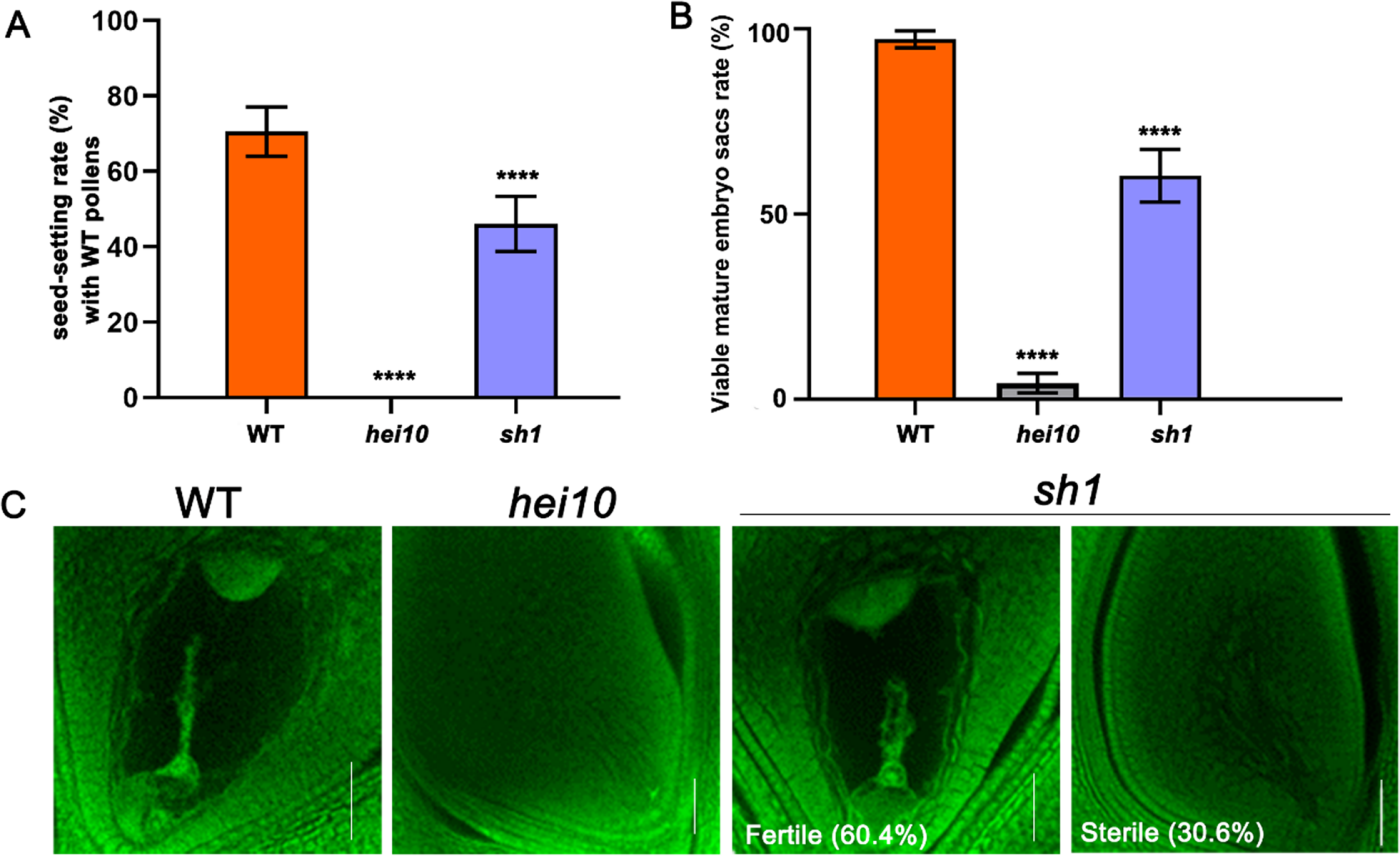
Female fertility is partially retained in the *sh1* mutant. **A** Seed setting rate of wild-type, *hei10*, and *sh1* panicles pollinated by wild-type pollen. For each line, 2 panicles in each of 5 independent plants were pollinated. **B** Embryo sac viability in wild-type, hei10, and sh1 lines. 50 embryo sacs from 5 independent plants were examined for each line. Data show mean ± SD. ****P ≤ 0.0001, two-tailed Student’s t-test. **C** Mature wild-type, hei10, and sh1 embryo sac. Scale bars = 50 μm

Unlike a previously reported *hei10* knockout allele that is completely male and female sterile (Wang et al. 2012), *sh1* retains partial female fertility. To explore *sh1* female fertility in more detail, homozygous *hei10* and *sh1* panicles were pollinated with WT pollen grains. We found that *hei10* spikelets (n = 288) could not set seed at all after pollination, while *sh1* spikelets (n = 319) exhibited ~ 46% seed setting compared with ~ 70% in WT lines (Fig. 5A). Subsequently, we monitored the development of WT, *hei10*, and *sh1* female gametophytes by embryo sac staining. Consistent with the seed setting results, the *hei10* embryo sacs (n = 250) did not develop normally at all, while 60.4% of *sh1* embryo sacs (n = 250) developed normally (Fig. 5B, C). Thus, while the *sh1* mutation completely disrupts male sterility, it has only moderate effects on female fertility.

**Fig. 6:**
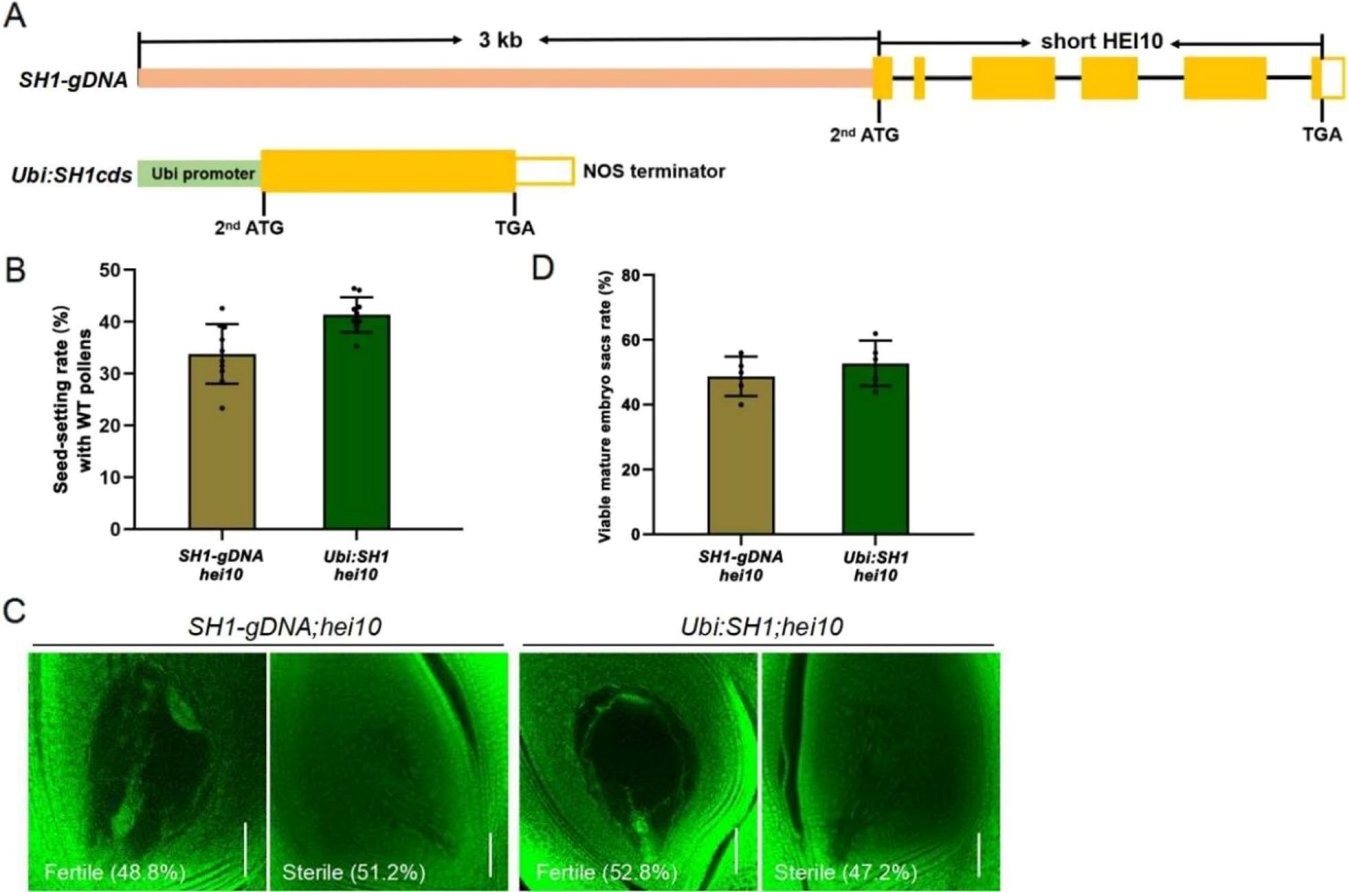
Recombinant short HEI10 partially restores female fertility in *sh1.* **A** Top: Schematic representation of the *SH1* locus containing the coding region of the presumed short *HEI10* and its 3 kb upstream sequence. Yellow boxes, *HEI10* exons 3–8. Bottom: Schematic arrangement of the maize ubiquitin promoter (Ubi) driving expression of the short *HEI10* coding sequence (CDS), terminated by a NOS terminator. The second start codon and stop codon of the *HEI10* CDS are indicated. **B** Seed setting rate of *SH1-gDNA;hei10* and *Ubi:SH1cds;hei10* transgenic panicles pollinated by wild-type pollen. For each line, 2 panicles in each of 5 independent plants were pollinated. **C** Mature embryo sacs from *SH1-gDNA;hei10* and *Ubi:SH1cds;hei10* transgenic plants. Scale bars = 50 μm. **D** Embryo sac viability in *SH1-gDNA;hei10* and *Ubi:SH1cds;hei10* transgenic lines. 50 embryo sacs from 5 independent plants were examined for each line. Data show mean ± SD

To explore whether the distinct impacts on male and female fertility in *sh1* were caused by the truncated sHEI10 protein or by changes in nearby sequences, we introduced the sHEI10 protein into *hei10* plants using two strategies. We used both the genomic *sh1* locus containing the coding region of the presumed sHEI10 and a 3 kb upstream sequence (*SH1-gDNA*); and an artificial construct where the maize *Ubiquitin* promoter was used to constitutively drive expression of *sHEI10* (*Ubi:SH1cds*; Fig. 6A). These constructs were individually transformed into homozygous *hei10* mutant plants, and expression of sHEI10 in the *SH1-gDNA:hei10* transgenic plants was confirmed with immunoblotting (Additional file 1: Fig. S7). Both transgenic lines exhibited similar phenotype as *sh1* with respect to seed-setting after pollination with WT pollen and the proportion of normally developed embryo sacs (Fig. 6B–D). These results suggest that the truncated sHEI10 protein is able to confer female fertility in *hei10* female-sterile lines, but cannot restore male fertility (Additional file 1: Fig. S8A).

### The RING domain of HEI10 is not required for its nuclear localisation and interaction with other meiotic proteins

**Fig. 7:**
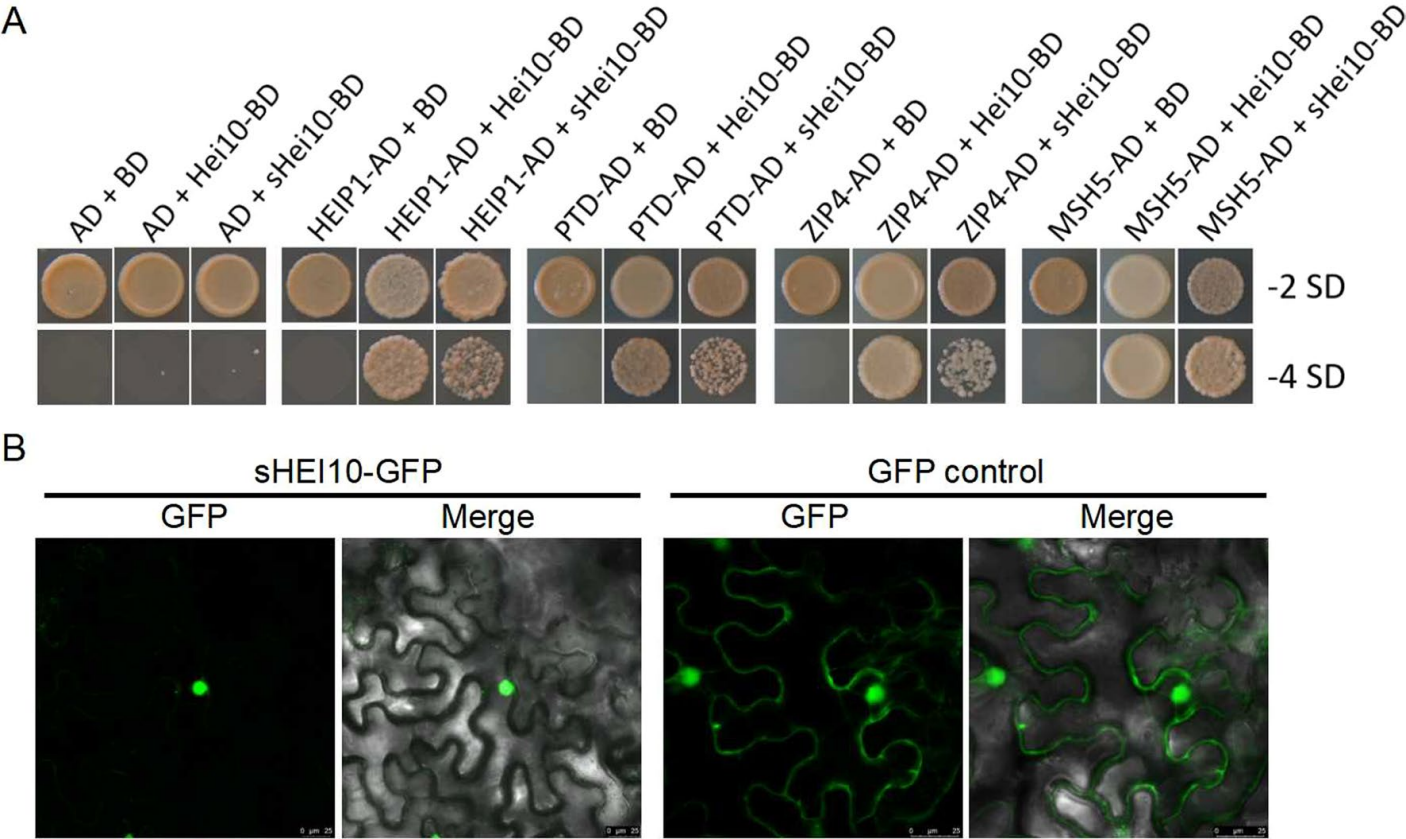
The RING domain of HEI10 is not required for nuclear localisation and interaction with other meiotic proteins. **A** Yeast two-hybrid assay of interaction between rice HEIP1, PTD, ZIP4, MSH5 and full-length HEI10 or short HEI10 (sHEI10). AD, activating domain; BD, DNA-binding domain; -2 SD, synthetic defined medium without leu and trp showing co-transformation of constructs; -4 SD, synthetic defined medium without leu, trp, ade, and his showing interaction of expressed proteins. **B** Subcellular localisation of short HEI10 tagged with GFP in tobacco leaf epidermal cells. “Merge” combines fluorescence and bright field images. Scale bars = 25 μm

A few ZMM proteins (HEIP1, PTD, ZIP4 and MSH5) have been reported to interact with HEI10 to co-regulate CO during rice meiosis; proper loading of HEIP1 requires HEI10, but not vice versa (Li et al. 2018; Zhang et al. 2019; Chang et al. 2020). A yeast two-hybrid (Y2H) assay confirmed that sHEI10 (lacking the RING domain) was able to interact with these ZMM proteins (Fig. 7A). Furthermore, GFP-tagged sHEI10 was able to localise correctly to the nucleus in tobacco leaf cells (Fig. 7B). We propose that the truncated sHEI10 protein remains partially functional in female gametes, enabling partial female sterility in *sh1* lines, while the RING domain remains essential for male fertility.

## Discussion

Correct functioning of meiosis-related genes is key for male and female fertility. In plants, HEI10 has a vital role in CO formation in both male and female gametophytes (Chelysheva et al. 2012; Wang et al. 2012). Here, we have found an allelic *hei10* mutant that expresses a truncated HEI10 protein lacking the N terminus RING domain, which is completely male sterile but retains partial female fertility.

Male and female meiosis in many species often exhibits a prominent difference in recombination rate (heterochiasmy). In Arabidopsis, CO numbers in male meiosis are 1.6-fold higher than in female meiosis, and the differences are thought to be regulated by both HEI10 dosage and SC length (Capilla-Pérez et al. 2021; Morgan et al. 2021; Durand et al. 2022). SC formation in Arabidopsis is regulated by ZYP1, mutation of which leads to increased CO number and erases heterochiasmy (Capilla-Pérez et al. 2021); while elevated expression level of HEI10 increases CO number in both male and female meiocytes (Durand et al. 2022). ZYP1 mutation in Arabidopsis does not obviously affect fertility, which makes it difficult to evaluate the influence of heterochiasmy on fertility. However, mutation of its homologous protein in rice, ZEP1, causes male sterility but normal female fertility (Liu et al. 2021), indicating that heterochiasmy and CO numbers might play more important roles in rice male fertility. The rice HEI10 null mutant displays complete male and female sterility (Wang et al. 2012), but sHEI10 lacking the RING domain retains partial female fertility (Figs. 5 and 6), implying that rice HEI10 participates in male and female meiosis differences and/or heterochiasmy, and that the RING domain is perhaps important in maintaining normal heterochiasmy for male fertility. Similar mechanisms may regulate crossover patterns in diverse eukaryotes.

HEI10 encodes a E3 ubiquitin ligase, containing a RING (Really Interesting New Gene) domain with C3HC4 structure. The RING domain normally acts as a protein interaction domain to recruit E2 ubiquitin-conjugating enzyme and directly transfers ubiquitin from E2 to substrate. The remaining part the RING-type E3 ligase contributes to the substrate recognition (Deshaies and Joazeiro 2009). The functions of HEI10 in fungal and mammalian species are well documented. In mammals, both HEI10 and its paralog RNF212 are required for the class I CO formation. They co-ordinately regulate the turnover of a subset of recombination factors by SUMO-dependent control of the ubiquitin-proteasome system (Reynolds et al. 2013; Qiao et al. 2014; Rao et al. 2017). In mice, RNF212 functions primarily as a SUMO ligase, which antagonizes the rate of HEI10 mediated substrate ubiquitination and destruction. On the other hand, HEI10 antagonizes RNF212 by promoting its proteasomal degradation (Reynolds et al. 2013; Qiao et al. 2014; Rao et al. 2017). In *Sordaria macrospora*, HEI10 integrates signals from the SC, associated recombination complexes, and the cell cycle to mediate both the development and programmed turnover/evolution of recombination complexes via SUMOylation/ubiquitination (De Muyt et al. 2014). Moreover, site mutation in the RING domain abolished E3 ubiquitin ligase activity of *Sordaria* HEI10, similar to the phenotype displayed by with HEI10 null mutant (De Muyt et al. 2014).

The structure of HEI10 proteins is highly conserved, but whether plant HEI10 proteins serve as E3 ligases and the function of their RING domains remain unclear. A recent study shows that ubiquitin localizes to the rice meiotic chromosomes, suggesting that ubiquitination is also involved in meiosis of plants (Li et al. 2018). Our results have demonstrated that the RING domain of rice HEI10 is not required for nuclear localisation and interaction with other ZMM family proteins such as HEIP1, PTD, ZIP4 and MSH5 (Fig. 7), but its function is indispensable for male meiosis (Fig. 3; Additional file 1: Fig. S5) and partially dispensable in female gamete development (Figs. 5 and 6). We speculate that this RING domain is essential in processes that direct recombination in male meiosis, but that normal HEI10 RING function, for example, E2 recruitment, may occur via other pathways in female gametes, where only the interaction of sHEI10 with ZMM proteins are necessary. However, the exact mechanism underlying partial female fertility in *sh1* lines, and whether the RING domain of HEI10 contributes to heterochiasmy, remain to be further studied. In future studies, the *sh1* mutant can be used to investigate the SUMO/ubiquitination profile of male and female gametes to reveal the regulatory roles of SUMOylation/ubiquitination during meiosis in two sexes. In addition, in vivo investigation of inter-relationships between known meiosis proteins with sHEI10 will provide clues to further understand the divergent roles of the RING domain in male and female meiosis.

## Conclusion

In this study, we demonstrate that missing the RING domain of HEI10 protein has different impacts on male and female meiosis as well as fertility in rice. Our results showed that the N terminus is not required for its nucleus localization and interactions with other recombination partners. We propose that meiotic proteins in rice male and female meiocytes may differ in the SUMOylation/ubiquitination, which is likely mediated by the RING domain of HEI10. Collectively, our results provide insights into the mechanisms of male and female meiosis diversities.

### Supplementary Information

**Additional file 1. Fig. S1**: Phenotype of *sh1* mutant.** Fig. S2**. Map-based cloning and analysis of *sh1* locus.** Fig. S3**. Chromosome behavior in male meiocytes is similar in *hei10* and *sh1* mutants.** Fig. S4**. *sh1* is a novel allele of *hei10*.** Fig. S5**. Female fertility is partially retained in the *sh1* mutant.** Fig. S6**. Recombinant short HEI10 partially restores female fertility in *sh1*.** Fig. S7**. The RING domain of HEI10 is not required for nuclear localisation and interaction with other meiotic proteins.** Fig. S8**. Male fertility of *SH1-gDNA;hei10* and *Ubi:SH1cds;hei10* transgenic plants.

**Additional file 2. Table S1**. Primers used in this study.

## Data Availability

All data supporting the findings of this study are available from the corresponding author on reasonable request.

## Abbreviations

SC: Synaptonemal complex
WT: Wild-type
*sh1*: *Shorter hei10 1*
MMCs: Microspore mother cells
SEM: Scanning electron microscopy
DAPI: 4′,6-diamidino-2-phenylindole
CO: Crossover
CRISPR-Cas9: Clustered regularly interspaced short palindromic repeats-associated protein-9
