# Supplementary material for: The RING Domain of Rice HEI10 is Essential for Male, But Not Female Fertility
Source: Rice (N Y). 2024 Jan 5;17:3. doi: 10.1186/s12284-023-00681-w (PMC10769960; doi:10.1186/s12284-023-00681-w)
Supplement: Supplementary file 1 — Additional file 1. Fig. S1: Phenotype of sh1 mutant. Fig. S2. Map-based cloning and analysis of sh1 locus. Fig. S3. Chromosome behavior in male meiocytes is similar in hei10 and sh1 mutants. Fig. S4. sh1 is a novel allele of hei10. Fig. S5. Female fertility is partially retained in the sh1 mutant. Fig. S6. Recombinant short HEI10 partially restores female fertility in sh1. Fig. S7. The RING domain of HEI10 is not required for nuclear localisation and interaction with other meiotic proteins. Fig. S8. Male fertility of SH1-gDNA;hei10 and Ubi:SH1cds;hei10 transgenic plants. [file 12284_2023_681_MOESM1_ESM.docx]

#### **The RING domain of rice HEI10 is essential for male, but not female fertility**

Qian Tan^a^, Xu Zhang ^a^, Qian Luo^a^, Yi-Chun Xu^a^, Jie Zhang^a^, Wan-Qi Liang^a^*

**Addresses:**

^a^Joint International Research Laboratory of Metabolic & Developmental Sciences, State Key Laboratory of Hybrid Rice, School of Life Sciences and Biotechnology, Shanghai Jiao Tong University, Shanghai, China.

*The corresponding author: Wan-Qi Liang

E-mail：[wqliang@sjtu.edu.cn](mailto:wqliang@sjtu.edu.cn)

# Supplementary DATA


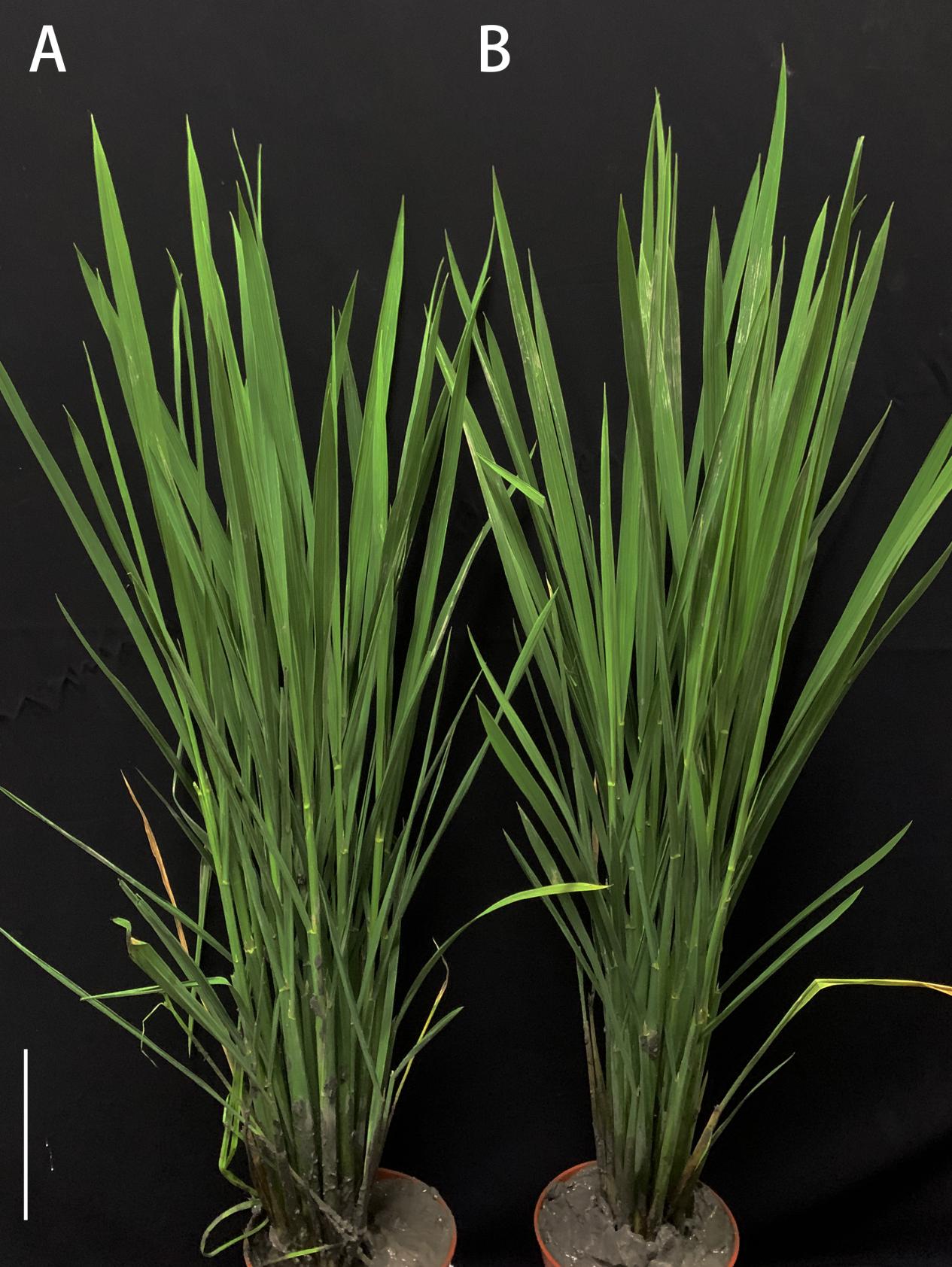


**Fig. S1. Normal vegetative growth of *sh1* plants**

A wild-type (A) and *sh1* (B) plant during vegetative growth. Scale bars = 10 cm.


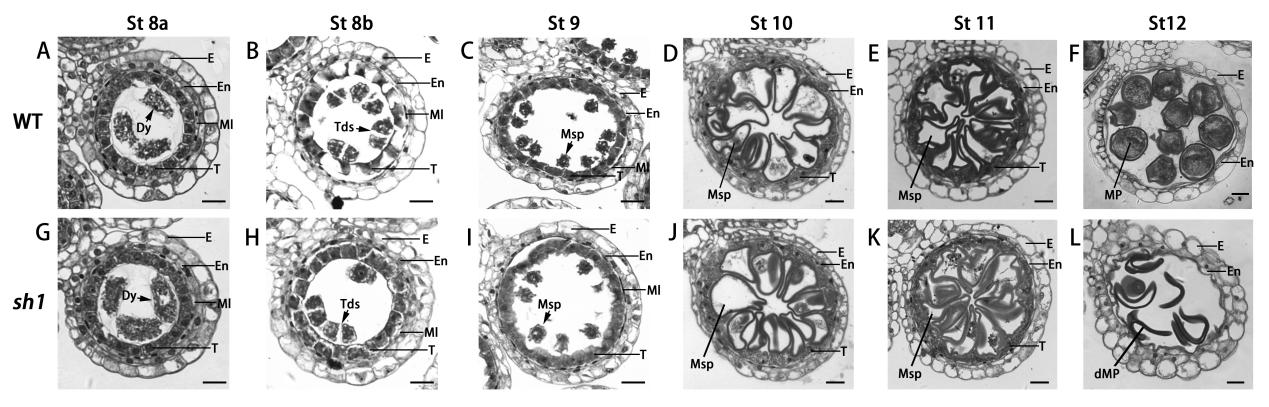


**Fig. S2.** Development of wild-type and *sh1* anthers*.*

Transverse section images of wild-type (A–F) and *sh1* (G–L) anthers from Stages (St) 8–12. Dy, dyad; E, epidermis; En, endothecium; MI, middle layer; Msp, microspore; dMP, degenerated microspores; MP, mature pollen; T, tapetum; Tds, tetrads. Scale bars = 25 μm.


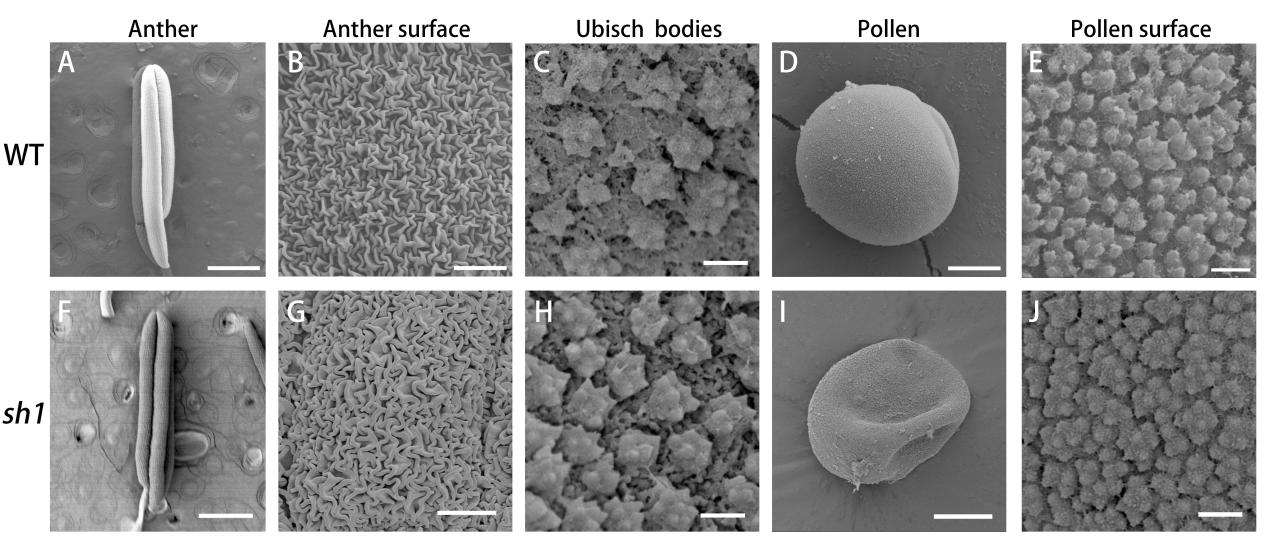


**Fig. S3.** Scanning electron microscopy (SEM) analysis of the surfaces of wild-type and *sh1* anthers and pollen grains at Stage 13.

(A,F) Wild-type (A) and *sh1* (F) anther. Scale bars = 40 μm.

(B,G) Wild-type (B) and *sh1* (G) anther surface. Scale bars = 5 μm.

(C,H) The inner surface of the wild-type (C) and *sh1* (H) anther wall. Scale bars = 1 μm.

(D,I) Wild-type (D) and *sh1* (I) pollen grain. Scale bars = 5 μm.

(E,J) Enlarged view of the wild-type (E) and *sh1* (J) pollen surface. Scale bars = 1 μm.


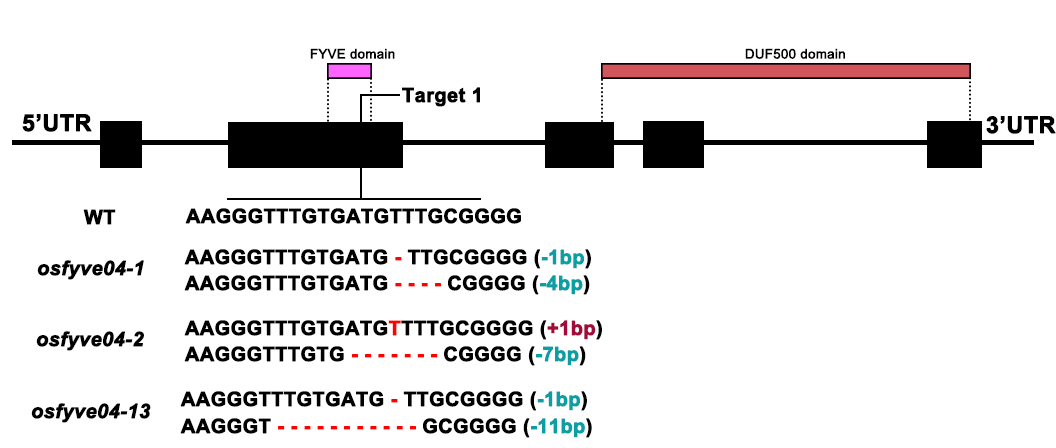


**Fig. S4.** CRISPR–Cas9-mediated targeted mutagenesis of *OsFYVE4*.

Schematic representation of the *OsFYVE4* gene, indicating the CRISPR–Cas9 target site, exons (boxes), introns (line), and untranslated regions (UTR). An alignment of wild-type (WT), *osfyve4-1*, *osfyve4-2*, and *osfyve4-13* sequences shows the biallelic mutations at the target site.


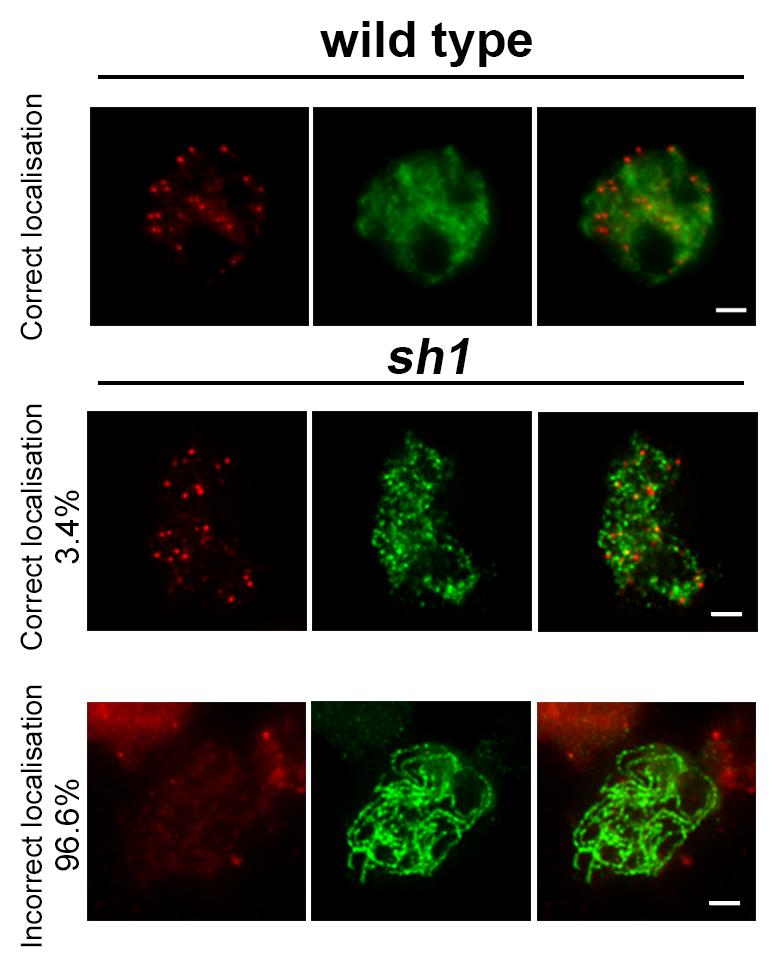


**Fig. S5.** HEI10 is not correctly localised in *sh1* mutant male meiocytes.

Co-immunolocalisation of HEI10 (red) and OsREC8 (green, a marker for meiotic chromosomes) in wild-type (n=24) and *sh1* mutant male meiocytes (n=379 cells). Scale bars = 5 μm.


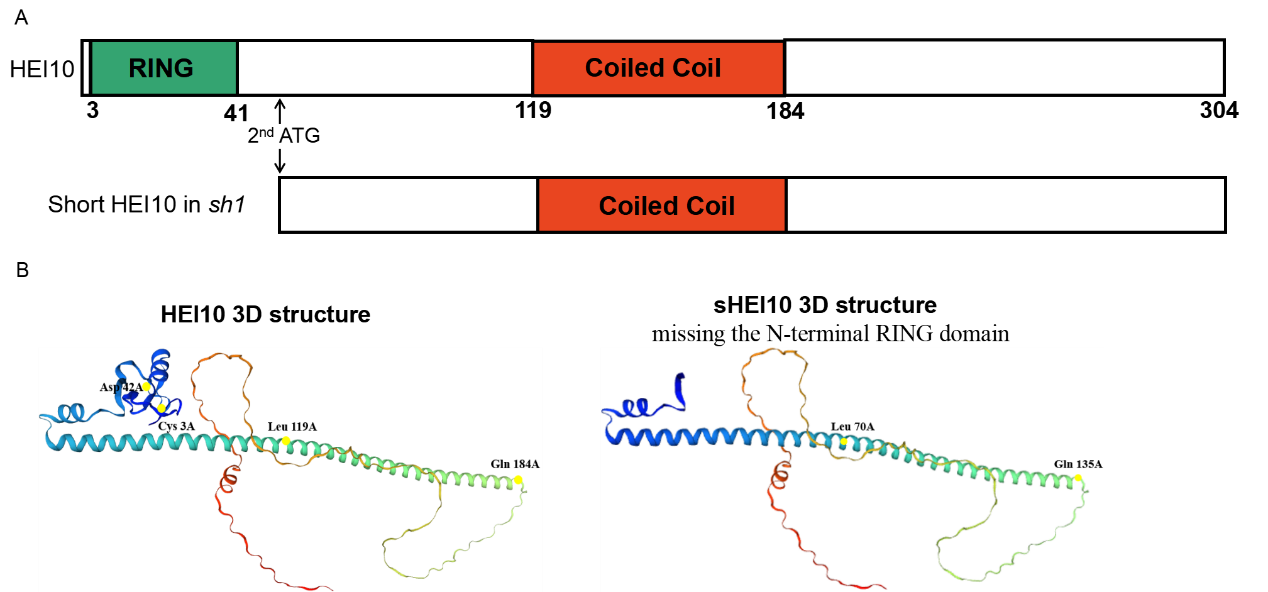


**Fig. S6. Functional domain and predicted protein structure of HEI10 proteins.**

(A) Schematic diagram of the wild-type rice HEI10 protein (up) and the truncated sHEI10 protein in *sh1* (bottom). The putative domains, amino acid numbers, and position of the second start codon are indicated.

(B) Comparison of HEI10 and sHEI10 protein structure. Predicted 3D protein structure of HEI10 (left) and sHEI10 (right) was modelled using SWISS-MODEL (https://swissmodel.expasy.org/). The secondary elements of the crystallographic structures are in rainbow colours, with N-terminus in blue, and C-terminus in red. HEI10 contains the N-terminal RING domain (amino acid residues position is Cys 3 to Asp 42), both proteins have the coiled-coil domain (Leu 119 to Gln 184 in HEI10, Leu 70 to Gln 135 in sHEI10).


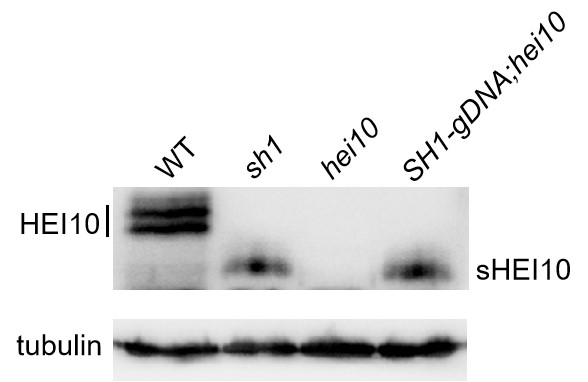


**Fig. S7.** Expression of full-length HEI10 or short HEI10 (sHEI10) in various plant lines.

Western-blot showing HEI10 and sHEI10 expression in wild-type (WT), *sh1* and *hei10* mutants, and *SH1-gDNA;hei10* transgenic lines. Tubulin is used as a loading control.


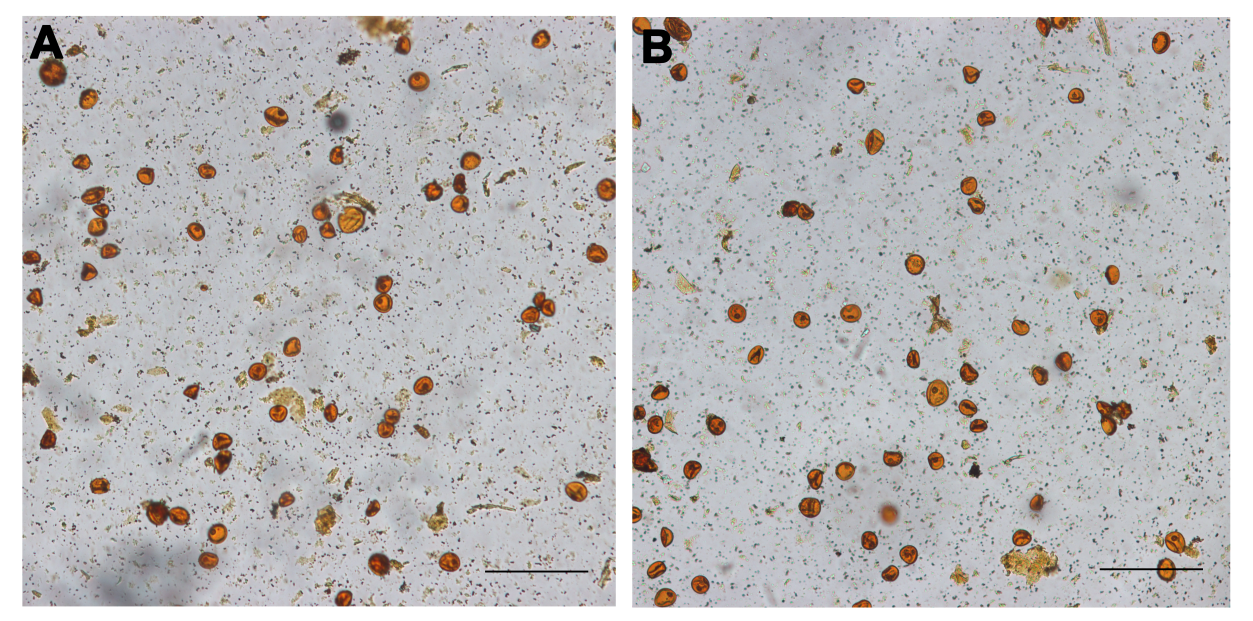


**Fig. S8. Male fertility of *SH1-gDNA;hei10* and *Ubi:SH1cds;hei10* transgenic plants.**

I_2_–KI staining of *SH1-gDNA;hei10* (A) and *Ubi:SH1cds;hei10* (B) mature pollen grains. Scale bars = 0.1 mm.
