# Supplementary material for: The RING Domain of Rice HEI10 is Essential for Male, But Not Female Fertility
Source: Rice (N Y). 2024 Jan 5;17:3. doi: 10.1186/s12284-023-00681-w (PMC10769960; doi:10.1186/s12284-023-00681-w)
Supplement: Supplementary file 2 — Additional file 2. Table S1. Primers used in this study. [file 12284_2023_681_MOESM2_ESM.docx]

**Table S1. List of primers used in this study.**

| **Primers** | **Sequence** |
| --- | --- |
| **Primers for map-based cloning** | |
| *Marker_C1_F* | ACGGCATGATGTTTCCATGACA |
| *Marker_C1_R* | ATGCTTCTTGATCCTCCGAGC |
| *Marker_C2_F* | TAGCACCCAACGAAACTGGAT |
| *Marker_C2_R* | GTAATAAGCTAGGGCCGGGAA |
| *Marker_C3_F* | AGGTGCAGAAATCGGGTGAG |
| *Marker_C3_R* | AAGATGGCATGTGCAGCTCT |
| *Marker_C4_F* | GTGAACTCCTTATTTGATGTCCTGT |
| *Marker_C4_R* | ACAGTTGTCAATAGTCAGTAAGCA |
| *Marker_C5_F* | AATATTTTGTTGGCGATGAGC |
| *Marker_C5_R* | ATGCACACCTTGAATGGACC |
| *Marker_C6_F* | CACACCACACGTGAATCTTGTC |
| *Marker_C6_R* | CGCCCAACAAGTCAATAACAGA |
| *Marker_C7_F* | CTCAGATCCACACGACAAGGG |
| *Marker_C7_R* | CATGCACGAGTGAGTGTGAGC |
| *Marker_C8_F* | TCAAAATTAGGTTTCGACCATCA |
| *Marker_C8_R* | TGAATACACGGAGAGAGCGAT |
| *Marker_C9_F* | GCGTACGGTTAATAACGAGACCA |
| *Marker_C9_R* | TGTACGGAACTTGACCCTCCA |
| **Primers for functional complementation** | |
| *HEI10gDNA-F* | CCGGGGATCCTCTAGAGAGGGAGCGTTGCCAT |
| *HEI10gDNA-R* | ATTCGAGCTGGTCACCTGGCCCGCTGCCATAA |
| **Primers for *sh1/hei10* biallelic lines identification** | |
| *ID-sh1-F* | TTTGTCTGTCGAATGCTGGTTC |
| *ID-sh1-R* | CGTTGCCTTGGTGGTGGAG |
| *ID-hei10-F* | ATGCTTGCTGGCGGGAGT |
| **Primers for *OsFYVE4* CRISPR knockout mutants** | |
| *FYVE04_Cri_F* | TAGGTCTCCGTGATGTTTGCGGTTTTAGAGCTAGAA |
| *FYVE04_Cri_R* | CGGGTCTCATCACAAACCCTTTGCACCAGCCGGG |
| *FYVE04_ID_F* | GGATATGGCCCAAACACTTGC |
| *FYVE04_ID_R* | AAGCATAAGCACATGATTTCTCAA |
| **Primers for *SH1-gDNA* and *Ubi:SH1cds* transgenic plants** | |
| ***SH1-gDNA-F*** | CGGTACCCGGGGATCCGGCAAACAGTAAAATT |
| ***SH1-gDNA-R*** | ATTCGAGCTGGTCACCTGGCCCGCTGCCATAA |
| ***Ubi:SH1cds-F*** | CGACTCTAGAGGATCCATGAGACCTGTTGATA |
| ***Ubi:SH1cds-R*** | GCTCTCTAGAACTAGTCTACAACGTGAACATA |
| **Primers for subcellular localisation analysis** | |
| *sHEI10-eGFP-F* | TCTCAAGCTTGGATCCATGAGACCTGTTGATA |
| *sHEI10-eGFP-R* | TGCTCACCATACTAGTCAACGTGAACATATGT |
| **Primers for Y2H assay** | |
| *BK-HEI10-F* | CATGGAGGCCGAATTCAAGTGCAATGCTTGCT |
| *BK-HEI10-R* | GCAGGTCGACGGATCCCTACAACGTGAACATA |
| *BK-sHEI10-F* | CATGGAGGCCGAATTCAGACCTGTTGATACAA |
| *BK-sHEI10-R* | Same as *BK-HEI10-R* |
| *AD-HEIP1-F* | GGAGGCCAGTGAATTCTGCATTGGCAGATTGT |
| *AD-HEIP1-R* | CGAGCTCGATGGATCCTTAAACACTTTTTGGA |
